# Enhancing epidemiological surveillance of the emergence of the SARS-CoV-2 Omicron variant using spike gene target failure data, England, 15 November to 31 December 2021

**DOI:** 10.2807/1560-7917.ES.2022.27.11.2200143

**Published:** 2022-03-17

**Authors:** Paula B Blomquist, Jessica Bridgen, Neil Bray, Anne Marie O’Connell, Daniel West, Natalie Groves, Eileen Gallagher, Lara Utsi, Christopher I Jarvis, Jo L Hardstaff, Chloe Byers, Soeren Metelmann, David Simons, Asad Zaidi, Katherine A Twohig, Bethan Savagar, Alessandra Løchen, Cian Ryan, Katie Wrenn, María Saavedra-Campos, Zahidul Abedin, Isaac Florence, Paul Cleary, Richard Elson, Roberto Vivancos, Iain R Lake

**Affiliations:** 1 COVID-19 Outbreak Surveillance Team, UK Health Security Agency, London, United Kingdom; 2 UK Health Security Agency, London, United Kingdom; 3 London School of Hygiene and Tropical Medicine, London, United Kingdom; 4 School of Environmental Sciences, UEA, Norwich, United Kingdom

**Keywords:** SARS-CoV-2, disease surveillance, s-gene target failure, COVD-19, public health, Omicron

## Abstract

When SARS-CoV-2 Omicron emerged in 2021, S gene target failure enabled differentiation between Omicron and the dominant Delta variant. In England, where S gene target surveillance (SGTS) was already established, this led to rapid identification (within ca 3 days of sample collection) of possible Omicron cases, alongside real-time surveillance and modelling of Omicron growth. SGTS was key to public health action (including case identification and incident management), and we share applied insights on how and when to use SGTS.

The emergence of the Omicron (Phylogenetic Assignment of Named Global Outbreak (Pango) lineage designation B.1.1.529) variant of severe acute respiratory syndrome coronavirus 2 (SARS-CoV-2) in November 2021 was of global concern because early estimates noted its short doubling time (1.5–3 days; [1]) and its potential for immune escape from vaccination (hazard ratios 1.86–4.32; [2]) and reinfection (5.4 times higher reinfection risk compared with Delta; [2]). Subsequent modelling studies indicated that in spite of reports of less severe disease (15–80% lower than the Delta variant (B.1.617.2) [3]), substantial numbers of hospitalisation could still result, presenting a challenge for health services. Rapid detection and monitoring was critical and S gene target surveillance (SGTS) was used for this purpose in the first few weeks following Omicron emergence. This study, conducted in England, aimed to assess the timeliness of SGTS in comparison with whole genome sequencing (henceforth referred to as sequencing [4]) to detect Omicron and to critique the public health utility of SGTS.

## Detection of SARS-CoV-2 Omicron in England

Following a positive SARS-CoV-2 PCR test, the gold standard for Omicron identification is sequencing. However, sequencing takes 8–14 days making it of limited use for rapid response (e.g. enhanced contact tracing; [5,6]). Worldwide, sequencing is technically, logistically and financially challenging and hence only a minority of coronavirus disease (COVID-19) samples are sequenced [4]. In England, around 10% of all PCR results positive for SARS-CoV-2 are sequenced. Some diagnostic assays target the S gene for SARS-CoV-2 detection, including the TaqPath reverse transcription PCR (RT-PCR) (Thermo Fisher, Waltham, United States) which targets three genes (N, ORF1ab, S). S gene target surveillance (SGTS) has proved a useful indicator of different variants. On the TaqPath assay, an undetectable S gene target is referred to as S gene target failure (SGTF) and is defined as quantification cycle (Cq) values ≤ 30 for N and ORF1ab targets but no detectable S gene target. An SGTF result is a sensitive indicator for Omicron, in particular the Omicron BA.1 lineage that emerged in November 2021 [7,8]. First use of SGTS as an indicator or screening method for variant surveillance was in December 2020, when SGTF was found to be an indicator for the Alpha variant (B.1.1.7), then again in April 2021, when a detectable S gene target (where all three targets have Cq values ≤ 30) was found to be an indicator for the emerging Delta variant. The target failure is caused by mutations in the S gene target: Both Alpha and Omicron genomes have a deletion corresponding to S protein positions 69 and 70 [7].

Assays with multiple gene targets (referred to as PCR genotyping) may be combined with SGTS to detect a range of variants ([5,9]), and this offers increased scalability, reduced cost and increased speed in comparison with sequencing [10]. Here we focus on SGTS, specifically the SGTF result as indicative of Omicron, because of its potential value as an accessible, rapid and accurate indicator for monitoring new variants. 

## Laboratory analysis and reporting

PCR testing for SARS-CoV-2 in England is undertaken at National Health Service and private laboratories. Four large facilities dedicated to SARS-CoV-2 PCR testing use the TaqPath assay and submit positive/negative results, alongside the Cq values of individual target results (i.e. SGTS) for surveillance [7], which represented 30–35% of all tests in England during late 2021. Around 10% of all PCR-positive samples are sequenced and priority is given to certain groups such as hospital patients and staff and international travellers [11]. The SGTS and sequencing results are distributed to local health protection teams for further investigations and public health actions. Following emergence of the Omicron variant in November/December 2021, this occurred daily.

## Guiding public health action

**Figure 1:**
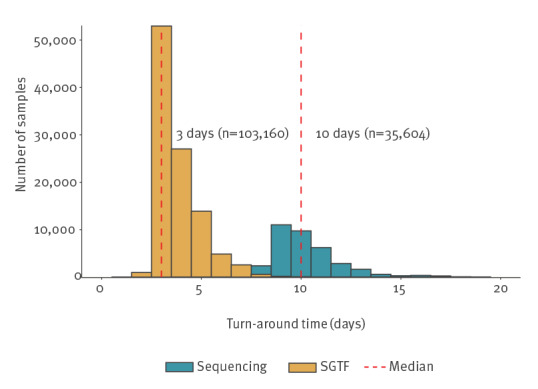
Turnaround time between specimen date and notification, COVID-19 screening, PCR vs sequencing, England, samples submitted 19–30 December 2021 (n = 138,764 samples)

Figure 1 presents the turnaround time of 103,160 cases with SGTF (from SGTS) and 35,604 cases with sequencing results from a 2-week period in December 2021. It was calculated as the difference between the specimen date (date when the sample was taken) and the date when the result was notified to local health protection teams. The SGTF results were available to health protection teams a median of 3 days (interquartile range (IQR): 3–4 days) after the specimen date, in comparison with sequencing results which were available a median of 10 days after (IQR: 9–11 days). This time advantage was critical as Omicron cases were initially doubling every 2 days. The SGTF method is faster as it is determined from the initial diagnostic test, whereas sequencing is a secondary or tertiary test and often involves transferring the sample to a large centralised facility.

COVID-19: coronavirus disease.

There is no reason to believe that samples from another time period would have produced a different result.

**Figure 2:**
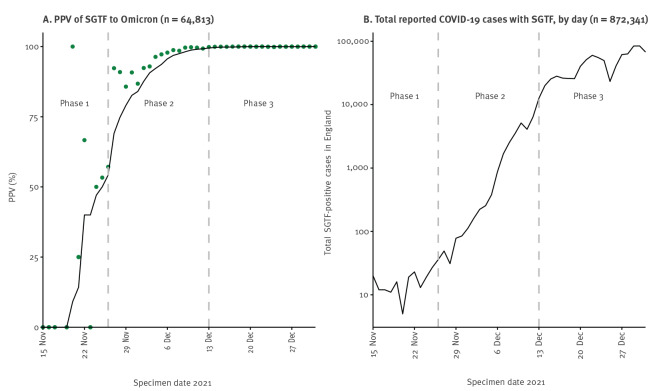
COVID-19 cases with S gene target failure, England, 15 November–31 December 2021 (n = 937,155)

COVID-19: coronavirus disease; PPV: positive predictive value; SGTF: S-gene target failure; SGTS: S gene target surveillance.

The utility of SGTS (specially the SGTF indicator) for public health is dependent upon the positive predictive value (PPV; [12]) of SGTF for Omicron, i.e. the proportion of samples containing SGTF confirmed as Omicron by sequencing (Figure 2A), the total SGTF cases (Figure 2B) and the coverage of SGTS (proportion of tests with SGTS). We describe how these guided public health action in three phases between mid-November and end-December 2021 (Table).

Panel A: points represent daily values, the line represents PPV of the past 7 days. Panel B: data are shown on a log scale. Public health action phases are described in detail in the Table.

**Table:** Public health action phases from S gene target surveillance for COVID-19, England, mid-November–end-December 2021

		Phase 1 Emergence	Phase 2 Rapid growth	Phase 3 Establishment
Sensitivity		High	High	High
PPV for Omicron		<50%	50-99%	>99%
Average daily cases with SGTF		<50	50–10,000	>10,000
Percentage of total cases with SGTF		<0.2%	0.2–50%	>50%
SGTF use	Case management	Yes	No (except in vulnerable settings)	No
	Modelling Omicron spread	No	Yes	Yes
Indicative dates in England (2021)		Mid- to late November	Late November to mid-December	From mid-December

PPV: positive predictive value; SGTF: S-gene target failure; SGTS: S gene target surveillance.

During Phase 1, the PPV of SGTF cases for Omicron was initially low, and few SARS-CoV-2 infections were caused by the emerging Omicron variant. The majority of samples with SGTF were understood to be Delta (consistently 0.01% of Delta cases had SGTF [13], which was probably due to low-quality samples in addition to true 69/70 deletions) and possibly small numbers of other variants with mutations in the S gene target [14]. Despite the frequent misclassification, SGTF was used in Phase 1 to identify possible Omicron cases, to target and slow establishment of this variant with public health action. This was considered acceptable as low numbers were still manageable and containing spread was a public health priority. For example, one case with SGTF (identified on 27 November 2021) triggered local enhanced contact tracing and deployment of mobile COVID-19 testing units [15], and did turn out to be Omicron. However, this was constrained by the fact that only 30-35% of cases had SGTS information. Daily SGTF case numbers were of limited use for modelling Omicron spread in the population because PPV at this time was low.

In Phase 2, cases with SGTF increased rapidly, which was consistent with ongoing community transmission that led to the establishment of this variant. Most sequenced SGTF specimens were Omicron variant and PPV rose to 99%. The use of SGTF for case management became impractical (except in vulnerable settings such as care homes) because of high numbers. The increased PPV made the SGTF data useful for modelling Omicron spread and associated severity (e.g. one of the first publications modelling Omicron spread using these data was published during Phase 2 on 11 December 2021; [16]). 

By Phase 3, daily cases with SGTF rose to more than 10,000 and nearly all cases with SGTF were Omicron. Monitoring shifted from cases with SGTF to all COVID-19 cases. Beyond Phase 3 from mid-January 2022 onwards, the proportion of cases with SGTF started to reduce again, coinciding with the increase in the Omicron BA.2 variant which has a detectable S gene [17]. 

## Ethical statement

Ethical approval was not required for this study as it was part of routine care/surveillance in England. Data were collected for contact-tracing and health protection purposes, falling under Regulation 3 of the Health Service (Control of Patient Information) Regulations 2002.

## Discussion

Use of SGTS enabled rapid identification of possible Omicron cases, with a median 3-day turnaround time. This was possible both because the emerging variant (Omicron) had a different S gene profile (i.e. was SGTF) to the dominant variant at the time (Delta), and because 30–35% of SARS-CoV-2-positive samples in England already had SGTS data collated.

Our experience is that well established data systems and flows are critical to the monitoring of all variants. For Omicron these specifically include data flow from SGTS and sequencing laboratories to centralised surveillance, as well as data flow from centralised surveillance to local health protection teams. It would be difficult to improve data flows mid-incident, especially with a rapid doubling time, therefore having established data systems in place is critical.

In England, it was helpful that SGTS was part of a wider surveillance strategy including other available detection technologies for SARS-CoV-2 variants. The SGTF indicator was valuable as an initial and rapid screening test but should be deployed alongside sequencing. Despite lower coverage and slower turnaround times, sequencing is necessary to detect new variants and should be conducted on a representative proportion of cases to monitor the accuracy of the SGTF indicator.

The role of genotyping [6] in Omicron surveillance was initially limited in England because of the lead-in time required to classify target assay combinations and the very rapid increase in cases. Specific assays for genotyping Omicron were unavailable until 15 December 2021. Once in place, however, genotyping became an important aspect of Omicron surveillance because coverage and timeliness (ca 4 days, data not shown) were similar and its ability to distinguish between variants was greater compared with the SGTF indicator.

Whichever assay is used, SGTS coverage is not 100% and varies geographically with implications for case management and surveillance interpretation. In some English regions, less than 20% of samples had established SGTS [7], and the coverage of this should be monitored over time and space to correctly interpret epidemiological trends.

## Conclusion

It is unclear why new SARS-CoV-2 variants have alternated between S gene target positivity and failure, or if they will in the future (e.g. Alpha with an undetectable S gene target, Delta with a detectable S gene target and Omicron BA.1 with an undetectable S gene target). This alternation has been essential for SGTS to detect an emerging variant against the backdrop of a pre-existing one. If and when it does occur, SGTS can play a critical role in guiding different phases of public health action. However this is also crucially dependent upon the surveillance system and methodologies being well prepared. SGTS remains of importance because it is able to distinguish between Omicron BA.1 (undetectable S gene target) and BA.2 (detectable S gene target).
